# Structure–Property Relationships in Bionanocomposites for Pipe Extrusion Applications

**DOI:** 10.3390/polym13050782

**Published:** 2021-03-04

**Authors:** Luigi Botta, Francesco Paolo La Mantia, Maria Chiara Mistretta, Antonino Oliveri, Rossella Arrigo, Giulio Malucelli

**Affiliations:** 1 Dipartimento di Ingegneria, Università di Palermo, Viale delle Scienze, 90128 Palermo, Italy; luigi.botta@unipa.it (L.B.); francescopaolo.lamantia@unipa.it (F.P.L.M.); mariachiara.mistretta@unipa.it (M.C.M.); 2 INSTM, Via Giusti 9, 50121 Firenze, Italy; Antonino.Oliveri@irritec.com (A.O.); rossella.arrigo@polito.it (R.A.); 3 IRRITEC, Via Gambitta Conforto, C.da S. Lucia SNC, 98071 Capo D’Orlando (Me), Italy; 4 Dipartimento di Scienza Applicata e Tecnologia, Politecnico di Torino, Viale Teresa Michel 5, 15121 Alessandria, Italy

**Keywords:** biopolymers, bionanocomposites, rheological behavior, mechanical properties, processability

## Abstract

In this work, bionanocomposites based on different biodegradable polymers and two types of nanofillers, namely a nanosized calcium carbonate and an organomodified nanoclay, were produced through melt extrusion, with the aim to evaluate the possible applications of these materials as a potential alternative to traditional fossil fuel-derived polyolefins, for the production of irrigation pipes. The rheological behavior of the formulated systems was thoroughly evaluated by exploiting different flow regimes, and the obtained results indicated a remarkable effect of the introduced nanofillers on the low-frequency rheological response, especially in nanoclay-based bionanocomposites. Conversely, the shear viscosity at a high shear rate was almost unaffected by the presence of both types of nanofillers, as well as the rheological response under nonisothermal elongational flow. In addition, the analysis of the mechanical properties of the formulated materials indicated that the embedded nanofillers increased the elastic modulus when compared to the unfilled counterparts, notwithstanding a slight decrease of the material ductility. Finally, the processing behavior of unfilled biopolymers and bionanocomposites was evaluated, allowing for selecting the most suitable material and thus fulfilling the processability requirements for pipe extrusion applications.

## 1. Introduction

Bionanocomposites are an emerging class of nanostructured hybrid biomaterials, involving a bioderived polymer combined with organic or inorganic fillers, showing at least one dimension at the nanometric scale [1,2,3]. These promising materials have potential industrial applications as an alternative to nanocomposites based on fossil-fuel derived thermoplastics, in attempting to solve the environmental concerns related to the intensive utilization of nonrenewable resources [4,5]. Similarly to the nanocomposites based on traditional polymers, the introduction of very low nanofiller loadings allows for enhancing some inherent properties of biopolymers, such as brittleness, low melt viscosity and low heat distortion temperature, which remarkably limit the utilization of these materials for a wide range of industrial applications [6,7,8]. Due to the wide number of biopolymer/nanofiller combinations, a large variety of bionanocomposites having tailored structural and/or functional properties and with application ranging from packaging [9,10] to agriculture [11] or automotive industry [12] can be designed and produced. Polylactic acid (PLA) represents the most used matrix for the formulation of bionanocomposites, due to its large utilization also at industrial scale [13,14,15,16]; nanoclays [17], graphene [18] and cellulose nanocrystals [19], among a few to mention, have been widely exploited as nanofillers for PLA-based systems, showing a great potential for the enhancement of the mechanical, optical, and barrier properties of this biopolymer.

The design of bionanocomposite materials having superior properties with respect to their unfilled counterparts is strictly related to the state of dispersion of the nanofillers within the host matrix [20]; In fact, nanofillers are usually prone to aggregate when dispersed in a viscous medium, and this issue significantly restricts the complete exploitation of their potential outstanding properties [21]. In addition, the aggregation phenomena hinder the achievement of a large polymer/nanofiller interfacial area, thus affecting the final properties of the resulting material [22]. Therefore, in aiming at obtaining a homogeneous dispersion of nanofillers, different strategies have been pursued, including the modification of the surface of nanofillers with functional groups that are able to enhance their compatibility with the biopolymeric matrices [23,24,25] and the use of different kinds of plasticizers and/or compatibilizing systems [13,16,26,27].

Furthermore, the selection of the proper processing method is of fundamental importance to obtain high-performance bionanocomposites. In fact, since the structuring of the material at nanoscale occurs during the processing step, the microstructure of the resulting material is determined at this stage [28]. Additionally, the processing conditions need to be optimized in order to ensure the achievement of a uniform morphology while preserving the structural integrity of the nanofillers and minimizing the possible degradation phenomena taking place the biopolymeric matrix [29]. 

Since melt mixing is one of the most exploited technologies for the formulation of bionanocomposites, especially at industrial level, the knowledge of the material’s rheological behavior under different flow regimes is mandatory for designing and modelling its processing and for optimizing the processing conditions [30]. In addition, the evaluation of the rheological response of nanostructured materials allows for inferring fundamental information about the state of distribution of nanofillers and the possible occurrence of polymer–filler and filler–filler interactions, being very sensitive to the modifications of the relaxation dynamics of macromolecular chains resulting from the incorporation of the nanofiller [31,32]. 

In this work, bionanocomposites based on different biodegradable polymers and two inorganic nanofillers, namely an organomodified clay and a nanosized calcium carbonate, were obtained through melt extrusion. The rheological and the mechanical properties of the obtained bionanocomposites were evaluated and compared to those of similar nanocomposites based on a traditional fossil-fuel derived polyolefin (namely HDPE). Furthermore, the processing behavior of the materials was thoroughly assessed, with the aim to assess the potential exploitation of the designed bionanocomposites for the production of irrigation pipes suitable for applications in agriculture. 

## 2. Materials and Methods

### 2.1. Materials

In this work, the following materials were selected as polymeric matrices:

- Bioflex F2110 (hereafter coded as BF) was obtained from FKuR Kunststoff GmbH (Willich, Germany) with the following properties: density = 1.27 g/cm^3^, MFI (190 °C, 2.16 kg) = 6 g/10 min. BF is a biodegradable and compostable polymer compound, certified as compostable according to EN13432 with a maximum thickness of 154 µm. The biobased carbon content (BCC), as reported in the technical data sheet, is 30%.
- MaterBi^®^ EF05B (hereafter coded as MB1) was obtained from Novamont (Novara, Italy) with these properties: density = 1.28 g/cm^3^, MFI (160 °C, 5 kg) = 3.5–4.5 g/10 min.
- MaterBi^®^ EF04P (hereafter coded as MB2) was obtained from Novamont (Novara, Italy) with these properties: density = 1.27 g/cm^3^, MFI (160 °C, 5 kg) = 4.5 g/10 min.

Furthermore, a high-density polyethylene (HDPE) Eraclene FB 506 from Versalis (Mantova, Italy) (density = 0.939 g/cm^3^, MFI (190 °C, 2.16 kg) = 0.2 g/10 min) was used as reference material.

Two kinds of nanofillers were used for the preparation of the nanocomposites, namely Cloisite 20A (hereafter coded as CL20A), which was obtained from Southern Clay Product (Rockwood Specialties, Inc., Gonzales, TX, USA) and is a ditallowdimethylammonium-modified montmorillonite with particle size <10 μm, and Socal 312 (hereafter coded as Socal), which was obtained from Solvay (Bollate, Milano, Italy) and is a nanosized calcium carbonate with a hydrophobic coating, having particles with a size of 50–100 nm. 

### 2.2. Nanocomposite and Pipes Preparation

The preparation of all investigated nanocomposites was carried out in a co-rotating twin screw extruder (OMC, Saronno, Italy); the processing conditions, i.e., temperature profile and screw rotation speed, were suited for each polymer matrix and are listed in Table 1. Prior to extrusion, either the polymers or the nanofillers were vacuum-dried: The former were treated at 60 °C for 4 h, while the CL20A and Socal were subjected to vacuum drying for 12 h at 120 and 90 °C, respectively.

Each nanofiller was added at 5 wt.% in the polymer matrix.

Specimens for rheological and mechanical characterizations were produced through a compression molding step, using a Carver laboratory press working at 100 bar; the temperatures selected for compression molding correspond to each single temperature achieved in the die during the extrusion process, namely 145 °C for nanocomposites based on MB1 and MB2, 175 °C for nanocomposites based on BF and 210 °C for nanocomposites based on HDPE.

In addition, nanocomposites based on BF and HDPE were subjected to a further extrusion step in a single screw (D = 19 mm, L/D = 25) extruder (Brabender, Germany) equipped with an annular die and a take-off unit. All the nanocomposites were extruded using a screw rotation speed of 80 rpm and the following temperature profile: 170-170-170-175 °C.

For comparison, unfilled polymers were subjected to the same processing.

### 2.3. Characterizations

The dynamic rheological behavior of unfilled matrices and all investigated nanocomposites was assessed through frequency sweep tests using an ARES G2 rheometer (TA instruments, New Castle, DE, USA) in parallel plate geometry (plate diameter = 25 mm; gap = 1 mm), from 10^−1^ to 10^2^ rad/s. The strain amplitude was fixed at 5%, which is within the linear viscoelastic region (as established from preliminary strain sweep tests).

Rheological characterization in shear flow was performed using a capillary rheometer Rheoscope 1000 (Ceast, Torino, Italy) with a capillary of D = 1 mm and L/D = 40. 

The same capillary rheometer, equipped with a tensile drawing unit, was exploited for assessing the rheological behavior of both the unfilled polymers and the nanocomposites in nonisothermal elongational flow. The melt strength (MS) and breaking stretching ratio (BSR) of the samples were measured; more specifically, MS refers to the force in the molten filament at break, while BSR is the ratio between the drawing speed at break and the extrusion rate. The temperatures selected for the rheological characterization of the different materials correspond to each single temperature achieved in the die during the extrusion process, as detailed in Section 2.2.

Mechanical analyses were performed on both compression molded samples and films using a Zwick/Roell dynamometer (Zwick/Roell Z005, Genova, Italy). An initial deformation speed of 1 mm/min was applied and maintained up to the achievement of 3.33% of deformation. Then, the speed was increased up to 100 mm/min until a break occurs. At least 5 specimens for each investigated system were tested, and the results were averaged.

The morphological characterization was performed through a SEM analysis, using a Philips ESEM XL 30 (Milano, Italy), on the fracture surfaces of gold-sputtered samples.

## 3. Results

### 3.1. Rheological Behavior

**Figure 1:**
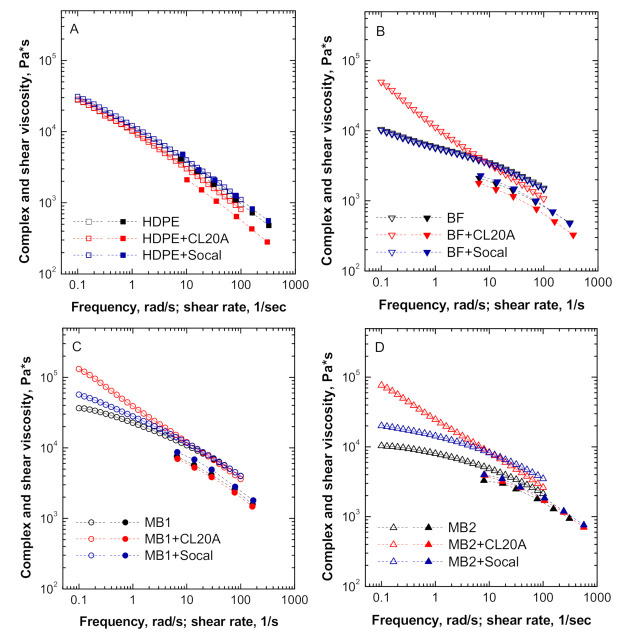
Complex and shear viscosity as a function of frequency and shear rate for all investigated materials: (**A**) HDPE-based systems, (**B**) BF-based systems, (**C**) MB1-based systems and (**D**) MB2-based systems.

Figure 1A–D shows the viscosities of all investigated materials, obtained by using both oscillatory and capillary rheometers. Firstly, it can be observed that apart from unfilled HDPE, all the materials (i.e., unfilled matrices and related nanocomposites) did not follow the Cox–Merz rule, which predicts the correspondence between the shear rate dependence of the shear viscosity and the frequency dependence of the complex viscosity [33]. This result can be explained considering that the Cox-Merz rule usually applies for linear homopolymers with a simple macromolecular architecture, while it fails for heterogeneous and multiphase materials, such as polymer blends and polymer-based nanocomposites [34,35].

As far as the trend of the complex viscosity is concerned, the rheological response of HDPE-based nanocomposites was almost unaffected by the presence of the nanofillers, indicating that a low degree of interaction between polyethylene macromolecules and embedded particles was achieved. Conversely, the bionanocomposites exhibited a different rheological response depending on the type of embedded nanofiller. More specifically, regardless of the biopolymer matrix, the introduction of Socal particles caused a slight enhancement of the complex viscosity values, when compared to the unfilled material, without significantly altering the trend of the viscosity as a function of frequency. This finding suggests that Socal particles are not able to promote substantial modifications of the macromolecular dynamics of the polymer macromolecules, according to similar results reported in the literature for composites containing nanosized calcium carbonate [36,37], and the rheological behavior of Socal-containing nanocomposites is mainly governed by the response of the respective matrices. On the other hand, CL20A-containing bionanocomposites exhibited a very different rheological response, involving the achievement of higher complex viscosity values with respect to their unfilled counterparts in the low frequency region. This finding indicates a remarkable effect of the embedded anisotropic nanofillers on the long-range dynamics of polymer chains. In particular, the apparent yield stress appearing at low frequencies can be associated with an arresting of the relaxation processes of biopolymer chains, due to the occurrence of polymer–nanofiller interactions, causing a restriction of the macromolecular dynamics. Furthermore, in the high frequency region, the effect of both Socal and CL20A nanofillers on the rheological response of bionanocomposites is relatively weak, indicating that the embedded nanofillers have a marginal effect on the short-range dynamics of biopolymer macromolecular chains.

Looking at the viscosity curves obtained through the capillary rheometer, the incorporation of Socal either kept the viscosity values unchanged or slightly improved them, compared to the unfilled matrices, at variance, nanocomposites containing CL20A exhibit lower viscosities than the respective unfilled polymers. This behavior can be explained considering the reduced resistance to the flow resulting from the possible orientation of the nanoclay layers or tactoids due to the convergent flow experienced by the materials at the entrance of the capillary [35]. 

**Figure 2:**
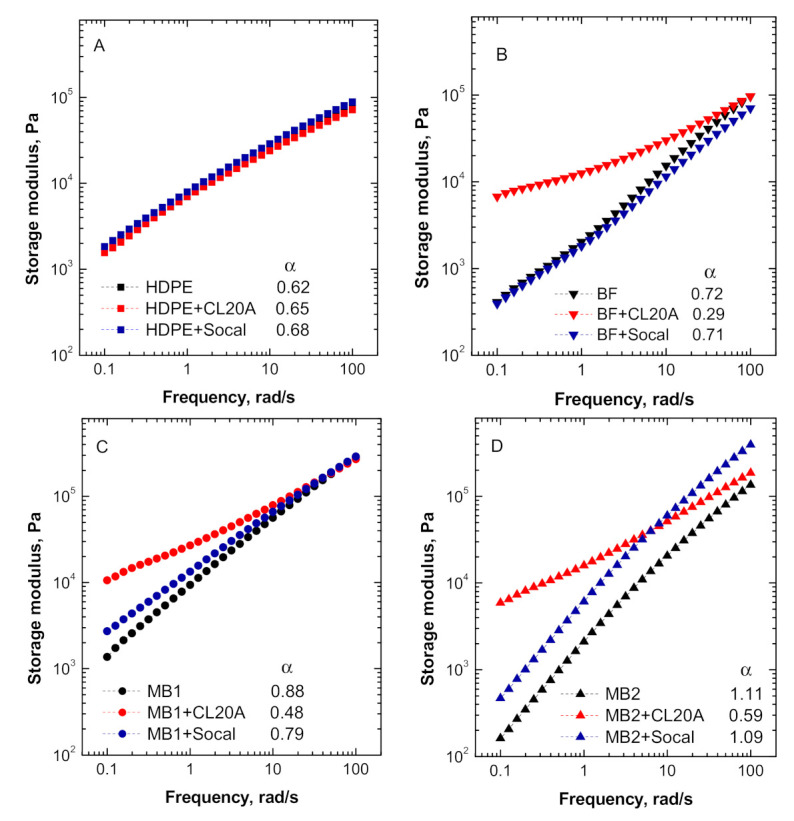
Storage modulus as a function of frequency for all investigated materials: (**A**) HDPE-based systems, (**B**) BF-based systems, (**C**) MB1-based systems and (**D**) MB2-based systems. In the insets, the values of the slope of the curves in the terminal region (α) are reported.

To further investigate the effect on the introduced nanofillers on the nanocomposite microstructure, the trend of the dynamic storage modulus (G′) as a function of frequency was evaluated and the results are shown in Figure 2A–D. 

As already inferred from the analysis of the complex viscosity curves, the introduction of both types of nanofillers within the HDPE matrix does not modify the rheological response of the host matrix. Furthermore, also in this case, all the bionanocomposites containing Socal particles show similar or slightly enhanced modulus values compared to the unfilled matrices, without remarkable changes of the G’ frequency dependence. Conversely, embedding the CL20A nanofiller induces a significant variation of storage modulus trend for all bionanocomposites, especially in the low frequency (terminal) region. More specifically, the modulus of CL20A-containing nanocomposites tends to become frequency-independent at low frequencies, indicating the occurrence of a transition from liquid-like to solid-like rheological behavior. To better investigate this behavior, the values of the slope of G’ curves in the terminal region (α) were calculated and reported in the insets in Figure 2. Usually, homogeneous polymers shows the so-called terminal behavior, implying a scaling behavior

(1) $$G^{\prime }\propto \;\omega^{2}$$

in the low frequency region, which is associated with the full relaxation of their macromolecular chains. It is important to highlight that all the investigated biopolymers exhibited lower slope values with respect to what was predicted from the linear viscoelasticity theory, due to their intrinsic multiphase heterogeneous structure; on the other hand, the low value showed by unfilled HDPE can be explained considering that the high molecular weight of this polymer, and the consequent high entanglement density, result in very long relaxation times [38]. For all the bionanocomposites containing the organomodified clays, the calculated α values clearly suggest the achievement of a nonterminal rheological behavior, implying the restraint of the long-range dynamics of polymer chains due to the establishment of polymer–filler interactions at the interface. More specifically, in the case of polymer-based nanocomposites containing layered silicates, this phenomenon can be associated with the formation of intercalated structures, as the confinement of the polymer chains in between the interlayer spaces slows down the motion of macromolecular chains, preventing their complete relaxation [39]. 

**Figure 3:**
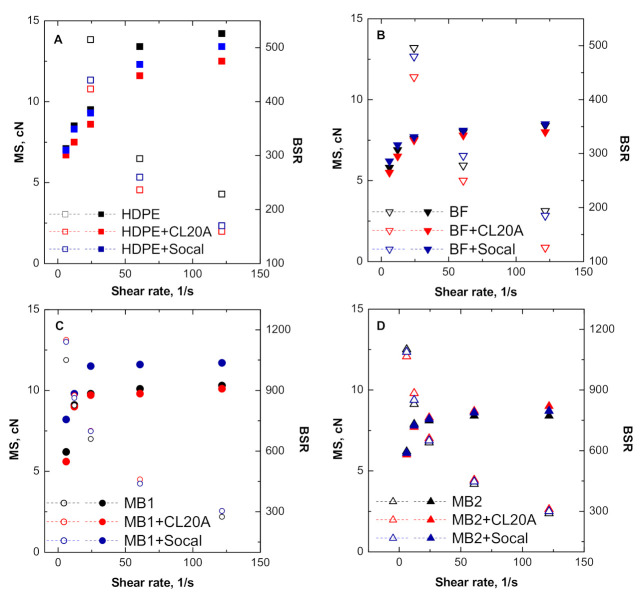
Melt strength (MS—filled symbols) and breaking stretching ratio (BSR—blank symbols) as a function of shear rate for all investigated materials: (**A**) HDPE-based systems, (**B**) BF-based systems, (**C**) MB1-based systems and (**D**) MB2-based systems.

To assess the processability of the formulated nanocomposites in processing operations involving a nonisothermal elongational flow, the assessment of their rheological behavior when subjected to this kind of flow is of fundamental importance. To this aim, the melt strength (MS) and the breaking stretching ratio (BSR) for all investigated materials were evaluated, and their variation as a function of the applied shear rate is presented in Figure 3A–D. 

Looking at the behavior of the unfilled polymers, HDPE shows the highest values of MS, according to the high viscosity of this material compared to the selected biopolymers. In addition, MB1 and MB2 exhibit an improved deformability with respect to HDPE, recognizable in the higher BSR values that were reached from these samples, while the stretchability of the BF material is quite similar to that of HDPE. 

As far as the nanocomposites are concerned, their general behavior reflects the previously discussed results about the shear viscosity, as usually higher values of MS are expected for the materials showing higher viscosity [40]. Interestingly, the HDPE + Socal nanocomposite exhibits a decreased MS with respect to the unfilled matrix, notwithstanding its slightly enhanced shear viscosity values. This behavior has already been observed for high-viscosity polymer melts, which experience the so-called “cohesive brittle melt rupture” during the application of the elongation flow [41]. This phenomenon implies a solid-like rupture with a minimum deformation at the break position and compromises the deformability of the melt due to the premature break of the material when compared to the typical liquid-like melt rupture. Therefore, in the case of HDPE + Socal, the melt is not able to reach the expected stress level due to its limited deformability, as also confirmed by the lower BSR values of the nanocomposite, compared to unfilled HDPE. 

Concerning the BSR values for the bionanocomposites, the introduction of both Socal and CL20A did not significantly affect the deformability of the biopolymers, suggesting the obtainment of a homogeneous dispersion of the nanofillers within the host matrices and a good level of polymer/filler interfacial adhesion.

### 3.2. Morphology

**Figure 4:**
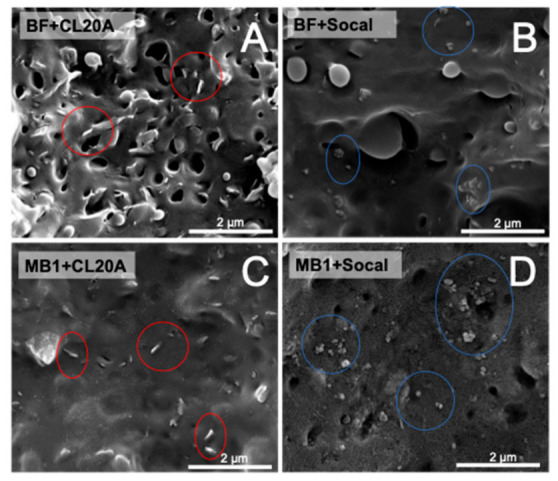
Typical SEM micrographs of bionanocomposites based on Bioflex F2110 (BF, (**A**,**B**)) and MaterBi^®^ EF05B (MB1, (**C**,**D**)).

The morphology of investigated materials was evaluated through SEM microscopy; Some typical micrographs of bionanocomposites based on BF and MB1 biopolymers are reported in Figure 4. Both the matrices show a complex morphology, and the presence of different polymeric phases is clearly observable. 

Regardless of the matrix, bionanocomposites containing CL20A exhibit a uniform dispersion of the embedded nanofillers, although either in BF- or in MB1-based systems, the organoclays seem to be prone to form tactoids, confirming the obtainment of intercalated structures already inferred from the analysis of the rheological behavior. Similarly, a good extent of nanofiller dispersion can be noticed in the Socal-containing bionanocomposites, notwithstanding the presence of some aggregates at the submicrometric scale.

### 3.3. Mechanical Properties

**Figure 5:**
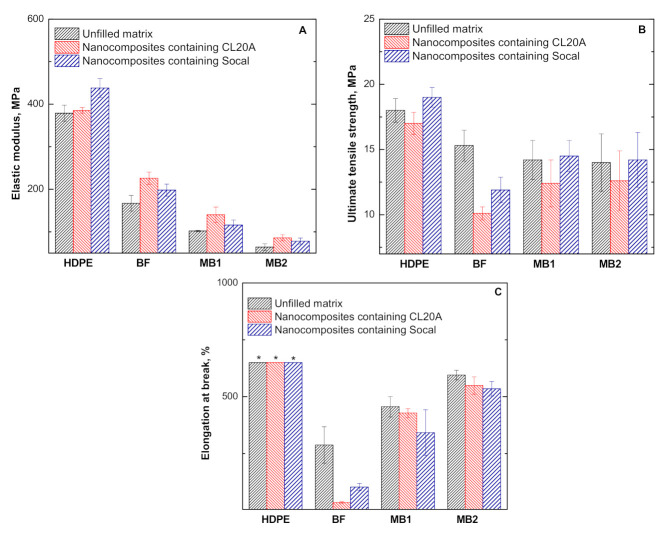
(**A**) Elastic modulus, (**B**) ultimate tensile strength, and (**C**) elongation at break for all investigated materials. Asterisk (*) indicates values exceeding the maximum measurable value, i.e., 630%.

The mechanical properties of all investigated materials were evaluated through tensile tests; the obtained results in terms of elastic modulus, ultimate tensile strength, and elongation at break are plotted in Figure 5A–C. As observable from the data presented in Figure 5A, the introduction of both Socal and CL20A caused an enhancement of the elastic modulus, which was not seen in the unfilled matrices, thus confirming the homogeneous dispersion of the embedded nanofillers within the matrices and the good extent of interfacial adhesion. Regarding the bionanocomposites, higher elastic modulus values were obtained for the CL20A-containing nanocomposites due to the presence of intercalated structures promoting an increase of the rigidity of the biopolymers. Looking at the values of ultimate tensile strength (Figure 5B), the nanocomposites based on HDPE, MB1, and MB2 containing Socal exhibit unchanged or slightly enhanced values when compared to the unfilled counterparts. At variance, the introduction of CL20A induces a decrease of the ultimate tensile strength values with respect to the unfilled matrices, especially in the case of BF-based nanocomposites. This finding can be associated with the premature failure of these nanocomposite samples, owing to their low ductility. In fact, the introduction of nanofillers induced a reduction of the elongation at break (Figure 5C), and this behavior is more pronounced for BF-based systems. The establishment of strong polymer/nanofiller interactions and the formation of intercalated structures in the case of CL20A-containing materials are responsible for the improved stiffness of the bionanocomposite samples, thus limiting their deformability and causing the premature breaking of the samples. 

### 3.4. Processing Behavior

The main purpose of this study was to assess the possible utilization of nanocomposite materials based on biopolymers as an alternative to traditional fossil-fuel based thermoplastics for the production of irrigation pipes. Therefore, the formulated materials were characterized through rheological and mechanical measurements aiming at selecting the most suitable material that meet the requirements, in terms of processability and mechanical properties, for pipe extrusion applications. 

**Figure 6:**
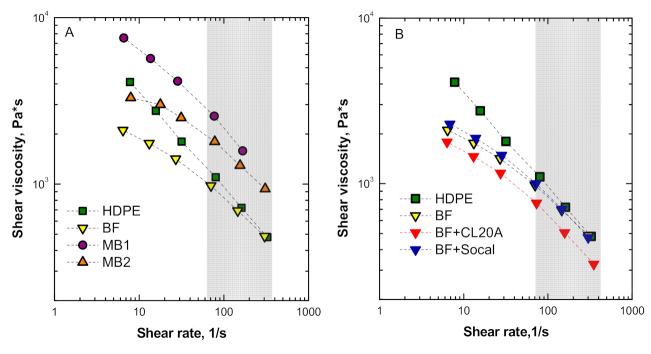
Shear viscosity as a function of shear rate for (**A**) unfilled matrices and (**B**) unfilled high-density polyethylene (HDPE) and BF and BF-based bionanocomposites.

To evaluate the processability of the exploited biopolymers, i.e., MB1, MB2, and BF, in comparison to that of the HDPE, which represents the “standard” material usually employed for pipe extrusion, it is useful to recall the rheological behavior of the polymers, in the typical shear rate range involved in extrusion operations. Figure 6A plots the shear viscosity curves of all unfilled polymers; the flow curves of HDPE and BF superimpose in the shear range interval of interest, while both MB1 and MB2 show higher viscosity values, indicating a different processing behavior with respect to HDPE. Interestingly, the introduction of Socal or CL20A did not significantly modify the processability of BF: In fact, as observable from the flow curves plotted in Figure 6B, the shear viscosity values of the bionanocomposites remain almost unchanged compared to those of the unfilled matrix. 

Based on these considerations, unfilled BF and BF-based nanocomposites were selected for the production of pipes, through a further processing step in a single screw extruder equipped with an annular die. Samples derived from the as-produced materials were then subjected to tensile tests to evaluate their mechanical behavior. Table 2 collects the obtained results, in terms of elastic modulus, ultimate tensile strength, and elongation at break, for BF and BF-based nanocomposites; the data are compared with those of standard HDPE subjected to the same processing.

From an overall point of view, all the selected materials exhibited a mechanical behavior similar to HDPE, indicating that the BF and BF-based nanocomposites also fulfill the requirements related to the mechanical performances.

Interestingly, the BF sample exhibited improved mechanical properties compared to the as-extruded material (the results are reported in Figure 5); the increase of the elastic modulus value can be attributed to the preferential orientation of the BF macromolecules along with the flow direction during the processing. Furthermore, the enhancement of the elongation at break can be explained by a modification of the material morphology that resulted from the elongational flow experienced by the biopolymer during the processing. More specifically, since BF is a polymer blend characterized by a multiphase microstructure, a possible variation of the morphology of the dispersed phase may occur, promoting an increased ductility [42]. 

A similar behavior was shown by the BF-based nanocomposites, as also in this case the samples exhibited enhanced mechanical performances with respect to as-extruded isotropic materials. The orientation of the macromolecules and of the organoclay nanofillers in the CL20A-containing system can be invoked to explain the rise of the elastic modulus values, while the obtained higher ductility can be associated with an improved nanofiller dispersion induced by the further processing step.

## 4. Conclusions

Bionanocomposites based on three different biopolymers and two types of nanofillers (i.e., Socal and CL20A) were formulated through melt extrusion, and the resulting materials were characterized through rheological, morphological, and mechanical analyses. 

Regardless of the biopolymeric matrix, the introduction of CL20A caused a significant modification of the material low-frequency rheological response, involving a slowdown of the relaxation processes of the biopolymer macromolecular chains due to the achievement of an intercalated morphology. By contrast, bionanocomposites containing Socal exhibited a rheological behavior quite similar to that of the unfilled matrices, notwithstanding higher viscosity values. The analysis of the high-shear rheological response suggests a marginal effect of the incorporated nanofiller on the trends of the shear viscosity curves; similar results were obtained from the characterization of the material behavior under nonisothermal elongational flow, as the presence of both Socal and CL20A did not remarkably affect the deformability of the biopolymers. 

The mechanical characterization pointed out a beneficial effect of the embedded nanofillers on the elastic modulus of the biopolymers, owing to the homogeneous dispersion of the nanofillers within the host matrices and the good extent of interfacial adhesion, notwithstanding a slight reduction of the materials ductility.

Finally, the processing behavior of all the investigated materials was evaluated with the aim to assess their suitability for the production of irrigation pipes. The analysis of the rheological behavior in the typical shear rate range involved in the extrusion processing allowed for identifying unfilled BF and BF-based nanocomposites as suitable systems, since they exhibited a similar processability compared to HDPE, which represents the “standard” material usually employed for pipe extrusion. Additionally, the mechanical characterization of the materials subjected to pipe extrusion indicated that BF and BF-based nanocomposites show adequate performances, thus confirming their possible exploitation for this specific industrial application as an alternative to traditional thermoplastic materials.

## Figures and Tables

**Table 1:** Processing conditions in the twin screw extruder for the formulation of all the nanocomposites.

Polymer Matrix	Temperature Profile [°C]	Screw Rotation Speed [rpm]
MB1 and MB2	90-110-130-140-140-145-145	200
BF	170-170-170-170-175-175-175	205
HDPE	130-150-170-190-190-200-210	200

**Table 2:** Main mechanical properties of the extruded pipes.

Sample	Elastic Modulus [MPa]	Ultimate Tensile Strength [MPa]	Elongation at Break [%]
HDPE	470 ± 15	35.7 ± 0.4	450 ± 7
BF	360 ± 26	26.7 ± 0.5	415 ± 5
BF + CL20A	450 ± 23	34.5 ± 0.9	423 ± 14
BF + Socal	380 ± 22	31.0 ± 1.0	373 ± 16

## Data Availability

The data presented in this study are available on request from the corresponding author.
